# Density and Habitat Relationships of the Endemic White Mountain Fritillary (*Boloria chariclea montinus*) (Lepidoptera: Nymphalidae)

**DOI:** 10.3390/insects8020057

**Published:** 2017-06-04

**Authors:** Kent P. McFarland, John D. Lloyd, Spencer P. Hardy

**Affiliations:** Vermont Center for Ecostudies, Norwich, VT 05055, USA; jlloyd@vtecostudies.org (J.D.L.); curlewsandpiper17@gmail.com (S.P.H.)

**Keywords:** *Boloria chariclea montinus*, White Mountain Fritillary, point count, distance sampling, butterfly survey methods, population monitoring, conservation, butterfly, Lepidoptera

## Abstract

We conducted point counts in the alpine zone of the Presidential Range of the White Mountains, New Hampshire, USA, to estimate the distribution and density of the rare endemic White Mountain Fritillary (*Boloria chariclea montinus*). Incidence of occurrence and density of the endemic White Mountain Fritillary during surveys in 2012 and 2013 were greatest in the herbaceous-snowbank plant community. Densities at points in the heath-shrub-rush plant community were lower, but because this plant community is more widespread in the alpine zone, it likely supports the bulk of adult fritillaries. White Mountain Fritillary used cushion-tussock, the other alpine plant community suspected of providing habitat, only sparingly. Detectability of White Mountain Fritillaries varied as a consequence of weather conditions during the survey and among observers, suggesting that raw counts yield biased estimates of density and abundance. Point counts, commonly used to study and monitor populations of birds, were an effective means of sampling White Mountain Fritillary in the alpine environment where patches of habitat are small, irregularly shaped, and widely spaced, rendering line-transect methods inefficient and difficult to implement.

## 1. Introduction

The White Mountain Fritillary (*Boloria chariclea montinus*) is a subspecies of the widely-distributed Arctic Fritillary (*B. chariclea*) and is endemic to the ca 1130 ha alpine zone of the Presidential Range of the White Mountains of New Hampshire, USA. This subspecies is a glacial relict that was likely widespread at the end of the last glaciation but that has since become isolated from conspecific populations as appropriate habitat in intervening areas vanished as the climate warmed. Based on reconstructions of post-glacial vegetation history, the alpine environment occupied currently by White Mountain Fritillary was isolated from retreating tundra vegetation by advancing subalpine forest approximately 9000 years before present [1]. Since then, White Mountain Fritillary has been isolated from populations that followed the retreating tundra northward.

Very little is known of the life history, habitat requirements, or abundance of White Mountain Fritillary. Although the conservation status of Arctic Fritillary is considered secure globally, White Mountain Fritillary is considered critically imperiled due to its perceived rarity and extremely limited distribution [2]. Scudder [3] (p. 605) offered the earliest comments on the population status:

“Probably no wandering collector has often seen more than eight or ten of these butterflies in a day’s scramble among the mountains, but if sought early in July they might be found in greater abundance; on a single occasion only I have seen as many as four at one time; they are most common about the steep heads of the great ravines”.

Later, Scudder [4] (pp. 152–153) wrote, “The ‘White Mountain Fritillary’ indeed seems really doomed to destruction. In the scanty numbers that one may find upon the mountain slopes, one sees the sign of their early departure; for, in the many years that I have searched for them with special pains, I have never seen more than a dozen or two specimens in a single day”. No systematic field surveys of White Mountain Fritillary have been conducted since, and so our understanding of this subspecies is only marginally better than it was over a century ago.

Effective conservation of White Mountain Fritillary, especially in light of threats to its alpine habitat from global climate change [5], requires a better understanding of its distribution, abundance, and population trends. In this study, we sought to provide the first description of distribution and density and to lay the groundwork for rigorous population monitoring by examining whether a commonly used approach for monitoring bird populations—point counts (i.e., surveys of a specified radial distance around a discrete point location)—could be used to survey White Mountain Fritillary.

## 2. Materials and Methods

We conducted this study in the Presidential Range of the White Mountains of New Hampshire, USA, in areas believed to be habitable by White Mountain Fritillary based on past observational records and museum specimens [6]. In particular, we focused on three alpine plant communities thought to support this species based on general surveys conducted in 2002 and 2003 [6]: heath-shrub-rush, herbaceous snowbank, and cushion-tussock (for descriptions of alpine plant communities, see [7]). These plant communities generally occur at elevations between 1250 m and 1850 m [7]. Heath-shrub-rush (115 ha) and cushion-tussock (94 ha) are the two most extensive plant communities in the alpine zone; herbaceous snowbank accounts for a very small fraction of the alpine zone (3 ha). Other mapped plant communities within the alpine zone that we did not survey because they apparently do not support White Mountain Fritillary included isolated patches of subalpine forest (krummholz and birch [*Betula*]—alder [*Alnus*] shrublands), sedge (*Carex bigelowii*) meadow, and fellfield.

We established 125 survey points in a GIS at random locations in the alpine zone (Figure S1), with the restriction that points had to fall within one of the three plant communities believed to support White Mountain Fritillary. We chose to use a point-based sampling approach, rather than line transects, because the plant communities thought to harbor White Mountain Fritillary occurred in small, fragmented, and irregularly shaped patches, and thus surveys conducted along line transects invariably sampled large areas that did not include our target plant communities. In establishing points, we deliberately undersampled cushion-tussock because we did not believe that this plant community supported large numbers of fritillaries. We sampled the other two plant communities with an intensity proportional to the area of each plant community. We censored 12 points because they could not be reached due to steep and rocky terrain, leaving a total of 113 points that were surveyed at least once in 2012 or 2013. Most points were in heath-shrub-rush (*n* = 103), with fewer in cushion-tussock (*n* = 4) and herbaceous snowbank (*n* = 6).

Because land managers allowed no permanent marking of survey points, we navigated to each using a handheld GPS unit. We visited each point 1–3 times during the flight time of White Mountain Fritillary. In 2012, we visited 37 points once, 28 points twice, 30 points three times, and a single point four times. In 2013, we visited 21 points once, 83 points twice, and one point three times. Logistic constraints imposed by inclement weather during the survey period prevented us from visiting every point in both years. Of the 113 survey points, we visited 88 at least once in both years, eight in 2012 only, and 17 in 2013 only. Across both years, we conducted 339 surveys at points in heath–shrub–rush, 11 at points in cushion-tussock, and 24 at points in herbaceous snowbank.

At each point, an observer conducted a 3-minute count, recording the distance to each butterfly observed in bands of 0−5 m, >5−10 m, >10−15 m, and 15–20 m. Butterflies can be easily alarmed by the approach of a human, and so we also counted any individuals that were driven from the count circle as the observer approached the point centroid. Surveys were not conducted during inclement weather (e.g., cloudy, fog, or rain) or high winds (Beaufort scale >4).

In addition to counting the number of White Mountain Fritillaries at each point, we also recorded information on environmental features that we thought might be important in explaining variation in abundance and in the probability of detecting butterflies that were present during our surveys. In particular, we recorded wind speed (estimated using the Beaufort wind-force scale) and the number of inflorescences within the count circle. We also obtained from the Mount Washington Observatory summit station the maximum daily temperature on each day that we conducted surveys.

We conducted all analyses using R 3.1.2 [8]. We estimated the mean number of inflorescences—an index of nectar resources—counted in the 20 m radius around each survey point (corresponding to the area surveyed for fritillaries) in each plant community and estimated a 95% confidence interval using a parametric bootstrap. For the bootstrap, we resampled counts with replacement, calculated a mean, and repeated 10,000 times; the 2.5 and 97.5 percentiles of the resulting distribution were used as the 95% confidence limits. We estimated the mean number of White Mountain Fritillaries per count and estimated a 95% confidence interval using the same parametric bootstrap.

However, raw counts of butterflies may yield biased estimates of abundance and density if not all individuals present within a survey area are detected during every survey. Distance sampling is one approach for estimating and correcting for this bias (e.g., [9]). Distance sampling assumes that the proportion of objects detected declines as a function of the distance between the object and the observer; with the further assumption that objects are distributed independently of the observer, which is achieved by random placement of survey points [10], we can use distance sampling to generate an estimate of the probability of detecting an object given that it was present during the survey $\left(\hat{p}\right)$. Dividing raw counts by $\hat{p}$ yields an unbiased estimate of the number of objects present within the survey area during the survey period. Distance sampling does not, however, produce an estimate of absolute abundance, because the estimate of detectability applies only to objects that are available for detection (i.e., objects that have a non-zero probability of being seen by the observer). Distance sampling will tend to overestimate $\hat{p}$ and thus underestimate abundance in cases where many of the objects of interest do not make themselves available for detection.

Hierarchical distance sampling [11] builds on this framework by allowing for the analysis of models that consider sources of variation in both detectability and abundance. Modeling the effect of covariates on detectability may produce more precise estimates of detectability, and modeling the effect of covariates on density can yield insights into the causes of spatial and temporal patterns of density. We used the R package “unmarked” [12] to apply hierarchical distance sampling to data collected during our point-count surveys of White Mountain Fritillary and to estimate density (individuals ha^−1^) of this species. We considered a variety of models containing different combinations of covariates for both detectability and abundance, and we used the small-sample correction of Akaike’s Information Criteria (AIC_c_) to determine which model we would use as the basis for inference. All continuous variables were standardized prior to analysis.

We began the modeling process by identifying the best combination of covariates of detectability. We suspected that observers differed in their ability to detect butterflies during surveys, and so we included observer identity in each of our models. Two observers conducted most (66%) of the surveys, with the remainder split among four other observers. Small sample sizes for these four observers made it difficult to estimate separate detection functions for each observer, and so we lumped them into a single group. Thus, observer became a categorical variable with three levels (the first two observers plus a third level consisting of the group of irregular observers). To this model, we added as covariates wind speed at the time of the survey and maximum temperature on the day that the survey was conducted. We did not consider models in which detectability varied among plant communities because sample sizes were inadequate in both cushion-tussock and herbaceous snowbank. All of these models were contrasted against two null models in which the detection function was described by a half-normal curve or hazard-rate curve with no covariates (i.e., detections vary only as a function of distance from the observer). The half-normal and hazard-rate function are both recommended by Buckland et al. [10] as useful, general models of a detection function.

Once we had identified the best-fitting model of detectability, we began adding covariates of density. The candidate set of models included all possible combinations (*n* = 15) of the following four variables (plus the covariates of detectability as selected in the previous step): year, because we noted substantial inter-annual differences in our raw counts and because we wanted to generate year-specific estimates of abundance; the week of the year in which the survey took place, under the assumption that abundance would vary as the flight season progressed; the number of inflorescences in the 20-m radius surrounding the survey area, as we believed that fritillaries might be more abundant in areas with more nectar resources; and the vegetation type in which the point was located, because previous observations [6] suggested that fritillaries were more common in herbaceous snowbank than in other alpine vegetation types. As in the analysis of detectability, we considered both the hazard-rate and half-normal key functions for each combination of covariates. Finally, because we suspected that our data might not follow a Poisson distribution (the default for distance-sampling models) due to the large number of counts where no fritillaries were detected, we re-ran the best-supported model with a negative-binomial distribution.

We used the best-fitting model from this candidate set as the basis for inference, and we tested its goodness-of-fit using a parametric bootstrap as implemented by the “parboot” function in the R package “unmarked”. In each step of this routine, the fitted model is used to generate a simulated dataset that meets the assumptions of the model, the model is refit, and the goodness-of-fit statistic is calculated (we used the Freeman–Tukey statistic). We repeated this process 250 times, and compared the observed value of the Freeman–Tukey statistic with the distribution of this same statistic generated by the bootstrap routine.

To examine the relationship between our estimate of detectability and its covariates, we calculated the expected detectability at each point based on the best-supported model (using the “getP” function in package “unmarked”) and calculated a mean and standard error at each level of the factor covariates. We estimated an approximate 95% confidence interval for each estimate as 2× standard error. We examined the relationship between maximum daily temperature, a continuous variable, and detectability using locally weighted scatterplot smoothing (LOESS).

We explored the relationship between predicted density and the covariates year and plant community by generating predictions from the best-supported model using a dummy dataset in which year and plant community varied but observer identity was held constant (set equal to Observer 1 for the purposes of illustration) and the number of flowers and maximum temperature were set to their mean value. We examined the relationship between predicted density and the number of flowers by generating predictions from the best-supported model using a dummy dataset in which year and plant community were held constant (with year set at 2012 and plant community set as heath-shrub-rush for the purposes of illustration), maximum temperature was held constant at the mean value, and the number of flowers varied across the range of observed values (excluding the highest 5% and the lowest 5% values, thus avoiding extreme values where predictive power was lowest). Although we examined only a subset of the possible predictions, the shapes of the predicted relationships illustrate general patterns in our findings because we did not consider models with interactions.

In these analyses, we treated visits to the same point in different years as independent replicates. We also treated repeated visits to a point within a year as independent replicates. We did so because the length of time between subsequent visits to a point within a year was relatively long (mean = 18 days; range = 4–33 days) and so we assumed that the populations sampled at each point were open to immigration, emigration, eclosure, and deaths during the interval between surveys. Thus, each visit should represent an independent trial for the purposes of estimating density and examining its covariates. However, if autocorrelation exists among repeated counts at a point within a year, then the precision of our estimates both of density and the effect of covariates on density will be overstated.

## 3. Results

We encountered White Mountain Fritillary less often and counted fewer individuals in 2013 than in 2012. We counted 102 White Mountain Fritillaries in 2012 during 190 surveys (mean count = 0.54; 95% CI = 0.33–0.77) and 40 during 184 surveys in 2013 (mean count = 0.22; 95% CI = 0.10–0.34) (Table S1, Figure S1). In 2012, we detected ≥1 individual during 51 of the surveys (26.8%) and in 2013 we detected ≥1 individual during 27 of the surveys (14.7%). Incidence of White Mountain Fritillary during a survey was not evenly distributed among plant communities: the vast majority of surveys in both cushion-tussock (90.9%; 10 surveys) and heath-shrub-rush (80.8%; 274 surveys) yielded no individuals, whereas 50% of surveys (*n* = 12) in herbaceous snowbank yielded ≥1 detection of White Mountain Fritillary. The maximum number of individuals counted during a survey in cushion-tussock was 1, but was 5 in herbaceous snowbank and 6 in heath-shrub-rush.

In 2012, we detected White Mountain Fritillary at 41 of the 96 points surveyed (42.7%); in 2013, we detected them at only 22 of the 105 points surveyed (21%; Table S1). Across both years, we detected fritillaries at only a single point in cushion-tussock (25% of points), at 42 points in heath-shrub-rush (41%), and at all six of the points in herbaceous snowbank. Consistency of counts within a point was relatively low: in 2012, 29 of the 41 points (70.7%) at which ≥1 individual was detected also had ≥1 survey in which zero individuals were detected, and in 2013 14 of the 22 points (63.6%) with ≥1 individual detected also had ≥1 survey in which zero individuals were detected.

The hazard-rate function best described the decline in detection as a function of distance from the observer, and detectability was influenced by the identity of the observer, wind speed during the survey, and maximum temperature on the day of the survey (Table 1). We used these combinations of covariates of detectability in each of the abundance models that we considered.

The best-supported model of abundance included effects of year, the number of inflorescences in the count circle, and the plant community in which the survey was conducted (Table 2). The goodness-of-fit for the model fit with the Poisson distribution was marginal (*p* = 0.07), but the same model fit with a negative-binomial distribution fit adequately (*p* = 0.50) and had a substantially lower AIC score (Table 2). Consequently, we used the negative-binomial model as the basis for inference.

**Figure 1:**
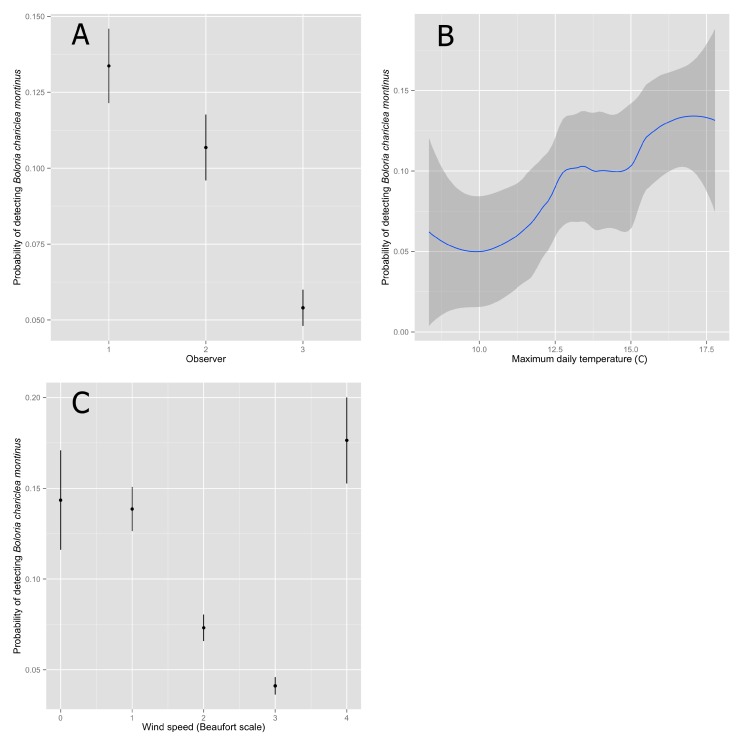
Estimated probability (mean ± 95% confidence interval) of detecting a White Mountain Fritillary (*Boloria chariclea montinus*) during a 3-minute point-count survey, given that it was present and available for detection, as a function of the observer (**A**), maximum temperature (Celsius) on the day of the survey (**B**), and wind speed during the survey (**C**). Surveys were conducted in 2012 and 2013 at 113 points in the alpine zone of the Presidential Range, New Hampshire, USA. Beaufort wind scale is 0 (calm; smoke rises vertically), 1 (light air; smoke drift indicates wind direction, still wind vanes), 2 (light breeze; wind felt on face, leaves rustle), 3 (gentle breeze; leaves and small twigs constantly moving, light flags extended), and 4 (moderate breeze; dust, leaves, and loose paper lifted, small tree branches move).

Based on the best-fitting model, detectability of White Mountain Fritillary was highest for Observer 1, slightly lower for Observer 2, and lowest for the group of irregular observers pooled together as Observer 3 (Table 3). Increasing wind speed in general had a negative effect on detectability (Table 3), although estimated detectability at the highest wind speed (category 4) was similar to detectability at the lowest wind speeds (categories 0 and 1). Maximum temperature had a significant, positive effect on detectability (Table 3). Overall, detectability estimates were low (Figure 1).

**Figure 2:**
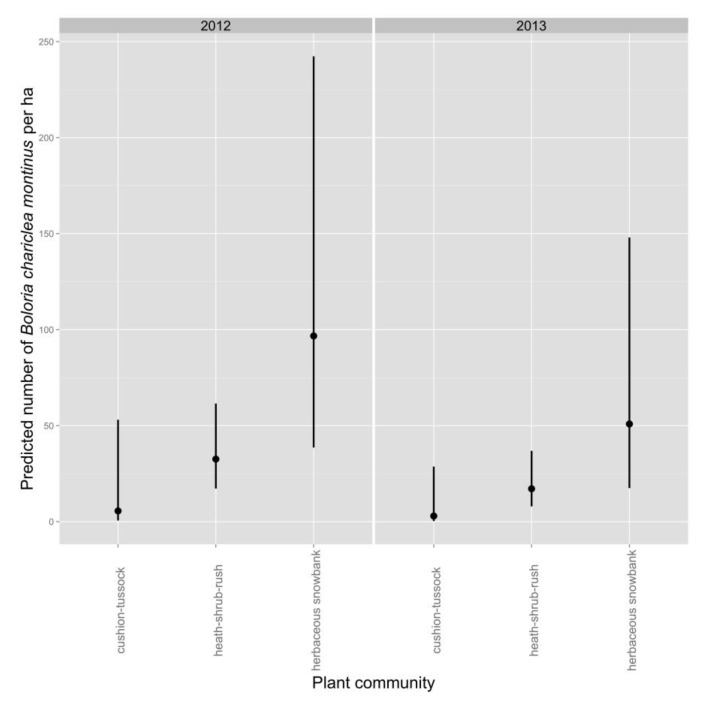
Predicted density (mean ± 95% confidence interval) of White Mount Fritillary (*Boloria chariclea montinus*) in different plant communities and in different years, as adjusted for detectability using distance sampling. Surveys were conducted in 2012 and 2013 at 113 points in the alpine zone of the Presidential Range, NH, USA.

**Figure 3:**
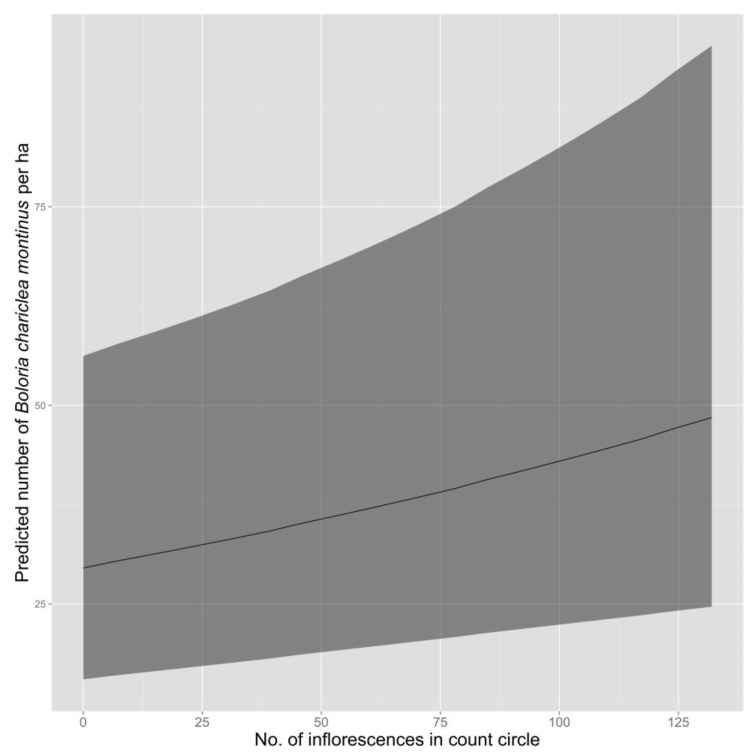
Predicted density (solid line = mean; shaded area = 95% confidence interval) of White Mount Fritillary (*Boloria chariclea montinus*), as adjusted for detectability using distance sampling, as a function of the number of inflorescences within a 20-m radius circle. Surveys were conducted in 2012 and 2013 at 113 points in the alpine zone of the Presidential Range, NH, USA.

Average density of White Mountain Fritillary during a survey was lower in 2013 than in 2012 (Table 4, Figure 2). Density during a survey was greatest in the herbaceous-snowbank community, intermediate in heath-shrub-rush, and lowest in cushion-tussock (Table 4, Figure 2). The best-supported model predicted an increase in density of fritillaries as the number of inflorescences in the count circle increased (Figure 3).

Although density of fritillaries varied in response to the number of inflorescences present, variation in the number of inflorescences did not appear to explain differences in fritillary density among plant communities. The number of inflorescences counted around survey points was similar in herbaceous snowbank (85.2; 95% CI = 67.1–104.7) and cushion-tussock (84.4; 95% CI = 70.7–98.2), despite substantial differences in density of fritillaries between these two communities, but was substantially lower in heath-shrub-rush (20.2; 95% CI = 14.4–26.9), which had intermediate densities of fritillaries.

## 4. Discussion

Enumerating the abundance of organisms, and understanding the causes of variation in abundance, is a central goal in conservation biology because doing so informs efforts to manage and conserve populations. The challenge of efficiently producing unbiased estimates of abundance for mobile organisms, such as butterflies, is not small, least of all because of the difficulty in accounting for individuals that go undetected during surveys. The problem of imperfect detectability has been recognized for many decades and has been addressed extensively in studies of birds (e.g., [9,13]), but methods for estimating detectability have been adopted in studies of butterflies only relatively recently [14,15,16]. Here, we have added to this growing body of literature by demonstrating that using point counts and distance sampling—a common strategy for monitoring bird populations—can be effective tools for studying and monitoring populations of butterflies. Indeed, the significant variation that we observed among observers in their ability to detect butterflies, as well as the effects of weather conditions on detectability, suggest that uncorrected counts of White Mountain Fritillary are unlikely to yield reliable estimates of abundance or density.

All of the studies of which we are aware that have applied distance sampling to the study of butterflies have relied on line transects. In general, line transects are more efficient than conducting surveys at point locations [10]. Point counts also run the risk of encouraging evasive movement, because the observer must walk through the survey area to arrive at the point location and in doing so they may disturb individuals prior to the onset of the count. Distance sampling conducted in the presence of non-random movement towards or away from the observer will lead to biased estimates of detectability. Despite this concern, our best-supported model fit the data adequately and thus we have no evidence of systematic violations of the assumptions of distance sampling during our point-count surveys.

However, point surveys may be more appropriate than line transects in some cases. For example, in the case of White Mountain Fritillary, individuals occur in small and irregularly shaped patches of habitat that are separated from one another by uninhabitable areas (e.g., extensive areas of lichen-covered rock, or fellfield), and so using line transects would involve either surveying large areas of non-habitat or using prohibitively short transects that remained within the boundaries of the habitat patch. Neither option is desirable. Point counts may also be useful in cases where traveling a line transect is difficult or unsafe, for example in very steep terrain, distracting the observer from the task of counting individuals and estimating distances. Our results suggest that point-count surveys can be employed to enumerate butterfly populations when such conditions exist.

In addition to demonstrating the efficacy of using point-count surveys to estimate density of butterflies, our study also represents the only systematic survey of the endemic White Mountain Fritillary and offers initial estimates of density for this subspecies. Although this subspecies has not vanished as predicted by Scudder [4] over a century ago, we found this species to be relatively uncommon and to occur at relatively low densities in most of the plant communities that we surveyed. Our results suggested that density was higher in the herbaceous snowbank community than in other alpine vegetation types inhabited by this taxon. Incidence of occurrence showed a similar trend: every point that we surveyed in herbaceous snowbank yielded ≥1 detection at some point during the study, whereas occurrence was much lower in the other plant communities. Herbaceous snowbank accounts for a very small area of the alpine zone (3 ha, or <1%) [6], and so even though density and occurrence is highest in this community the total number of adult fritillaries is likely far greater in heath-shrub-rush, which covers approximately 115 ha of the alpine zone [6]. The cushion-tussock community is also fairly extensive (94 ha) in the alpine zone of the Presidential Range but appears to be used only sparingly by adult White Mountain Fritillary.

Why density and frequency of occurrence is higher in herbaceous snowbank than in other community types is unknown. Overall, density of White Mountain Fritillary during a survey increased as the number of inflorescences in the count circle increased, presumably due to increased availability of nectar resources. However, the mean number of inflorescence counted around each survey point was similar in herbaceous snowbank and cushion-tussock, yet cushion-tussock had the lowest estimated density of fritillaries, so inflorescence density alone cannot explain the variation in density of fritillaries among plant communities. The herbaceous snowbank community, which appears generally wetter than other communities, may support larval host plant species or preferred species for nectaring that are absent or occur at lower density in other communities. However, these hypotheses cannot be tested because nectar preferences and the larval host(s) for White Mountain Fritillary remain unknown.

We also found substantial inter-annual differences in density, which could reflect stochastic variation caused by variable weather conditions or perhaps a biennial adult phenology such as that found in some more northern subspecies [17].

The populations of White Mountain Fritillary that we sampled at each survey point were demographically open between surveys, and thus our estimates of density are best considered as instantaneous measures of the number of fritillaries using the survey area at the time of the survey. Although our estimates could be expressed as abundance, summing estimates across surveys would not yield a reliable estimate of population size because our repeated counts at a point include an unknown mixture of individuals that may have been previously counted and individuals that were not available for detection during prior surveys (because, e.g., they had not yet eclosed or because they immigrated during the interval between surveys). Future efforts to estimate population size of White Mountain Fritillary—an important goal given the presumably small number of individuals in this population and threats to their habitat such as climate change—would benefit from better information on natural history and demographics (e.g., adult life span), and by adopting survey methods better suited to an open population. For example, increasing the number of visits to a point, and ensuring that some of the repeated counts were conducted at intervals short enough that the population could reasonably be considered closed, would allow for the application of open-population models such as that of Dail and Madsen [18]. We explored using these models on our dataset, but in initial analyses found that our relatively small sample size and lack of closed periods precluded their use. Future efforts at population monitoring will therefore require an increase in the extent and intensity of surveys, and the results presented here may be useful in more efficiently allocating effort.

Our results should also be viewed with caution because we did not directly account for the repeated nature of our counts. Distance sampling models implemented under a frequentist paradigm do not currently allow for the inclusion of random effects, which would be one means of addressing this problem. We found little consistency among counts at a point, yet if spatial auto-correlation in density of fritillaries exists then the precision of the estimated effects of covariates will be overstated because our sample size was inflated by treating each visit to a point as an independent replicate. This is apt to be most problematic when considering the effect of plant community on density, as this covariate was constant across surveys within a point, and least problematic when considering the effect of the number of inflorescences, which varied from survey to survey.

## 5. Conclusions

Our results suggested that adult White Mountain Fritillaries used primarily the herbaceous snowbank and heath-shrub-rush communities, and thus these communities should be the target for conservation and future monitoring efforts. Monitoring efforts should weight survey effort towards the two most frequently used plant communities, and a relatively lower level of effort should be directed at the cushion-tussock community, which does not appear to be an important habitat for White Mountain Fritillary. We have shown that point counts are a useful approach for surveying in the patchy environment that comprises the alpine zone in the Presidential Range, but we suggest adopting methodologies that account for imperfect detectability while also addressing the demographically open nature of the populations sampled. Finally, future research that verifies our findings of habitat-specific variation in density, and elucidates its causes, perhaps in concert with the collection of more information on natural history, would prove useful in developing conservation strategies that would allow this endemic legacy of past environmental change to persist in the face of present-day global change.

## Figures and Tables

**Table 1:** Comparison of models of detectability of White Mountain Fritillary counted at survey points during 2012 and 2013 in the alpine zone of the Presidential Range, New Hampshire, USA.

Covariates	Key Function	No. Parameters	AIC ^1^	ΔAIC
Observer+ wind speed + temp.	Hazard-rate	10	940.12	0
Observer + wind speed	Hazard-rate	9	961.43	21.32
Observer + wind speed + temp.	Half-normal	9	964.32	24.21
Observer + wind speed	Half-normal	8	977.65	37.53
Observer	Hazard-rate	5	979.78	39.66
Observer	Half-normal	4	988.13	48.01
	Hazard-rate	3	998.26	58.15
	Half-normal	2	1000.39	60.27

^1^ AIC = Akaike’s Information Criteria.

**Table 2:** Comparison of models^1,2^ explaining variation in abundance of White Mountain Fritillary counted at survey points during 2012 and 2013 in the alpine zone of the Presidential Range, NH, USA.

Covariates	No. Parameters	AIC	ΔAIC
Year + no. inflorescences + plant community (negative binomial)	17	797.01	0
Year + no. inflorescences + plant community (Poisson)	16	879.85	82.84
Year + no. inflorescences + plant community + week	17	881.84	84.83
Plant community	16	871.86	100.89
Plant community + week	15	872.68	101.41

^1^ Twenty-six other models with ΔAIC >105 were considered but are not shown here. ^2^ Each of the best-supported models was fit with the hazard-rate function; none of the models using the half-normal function were supported by the data.

**Table 3:** Parameter estimates and statistical significance of detectability parameters from the best-supported model of White Mountain Fritillary density and detectability. Data were collected during point-count surveys conducted in 2012 and 2013 in the alpine zone of the Presidential Range, NH, USA.

Parameter	β	SE	Z	*p*-Value
Intercept	1.774	0.442	4.02	<0.001
Observer 2	−0.389	0.193	−2.02	0.043
Observer 3	−0.677	0.192	−3.54	<0.001
Wind speed 1	−0.089	0.307	−0.29	0.772
Wind speed 2	−0.569	0.335	−1.70	0.089
Wind speed 3	−0.984	0.367	−2.68	0.007
Wind speed 4	−0.194	0.373	−0.52	0.603
Temperature	0.190	0.076	2.51	0.012

**Table 4:** Parameter estimates and statistical significance of parameters from the best-supported model of density of White Mountain Fritillary. Data were collected during point-count surveys conducted in 2012 and 2013 in the alpine zone of the Presidential Range, NH, USA.

Parameter	*β*	SE	Z	*p*-Value
Intercept	1.718	1.150	1.49	0.135
Year 2013	−0.643	0.280	−2.29	0.022
No. inflorescences	0.292	0.104	2.80	0.005
Heath–shrub–rush	1.766	1.107	1.60	0.111
Herbaceous snowbank	2.854	1.138	2.51	0.012

## Supplementary Materials

The following are available online at http://www.mdpi.com/2075-4450/8/2/57/s1, Table S1: Comma delimited text file of butterfly count data and covariates. Figure S1: Large format map (JPEG) of the alpine zone vegetation communities [7], hiking trails, and White Mountain Fritillary survey points with high count indicated.
